# Positive and negative regulation of Shh signalling in vertebrate retinal development

**DOI:** 10.12688/f1000research.16190.1

**Published:** 2018-12-14

**Authors:** Viviana Gallardo, Paola Bovolenta

**Affiliations:** 1 Centro de Biología Molecular , CSIC-UAM, Madrid, 28049, Spain; 2 CIBER de Enfermedades Raras (CIBERER), Madrid, 28029, Spain

**Keywords:** Cell cell communication, Shh Signalling, retina, development, eye, patterning, regulation

## Abstract

Cell-to-cell communication is fundamental for embryo development and subsequent tissue homeostasis. This communication is often mediated by a small number of signaling pathways in which a secreted ligand binds to the surface of a target cell, thereby activating signal transduction. In vertebrate neural development, these signaling mechanisms are repeatedly used to obtain different and context-dependent outcomes. Part of the versatility of these communication mechanisms depends on their finely tuned regulation that controls timing, spatial localization, and duration of the signaling. The existence of secreted antagonists, which prevent ligand–receptor interaction, is an efficient mechanism to regulate some of these pathways. The Hedgehog family of signaling proteins, however, activates a pathway that is controlled largely by the positive or negative activity of membrane-bound proteins such as Cdon, Boc, Gas1, or Megalin/LRP2. In this review, we will use the development of the vertebrate retina, from its early specification to neurogenesis, to discuss whether there is an advantage to the use of such regulators, pointing to unresolved or controversial issues.

## Introduction

The highest functions of the nervous system are based on communication among the huge variety of cells that compose the vertebrate brain. Communication among cells is also fundamental for correct development of the nervous system. Although there are several ways in which neural cells (and cells in general) exchange information, communication mediated by families of signaling molecules such as Wnt, bone morphogenetic protein (BMP), fibroblast growth factor (FGF), and Hedgehog (Hh) is one of the most common. These molecules activate specific signaling pathways that share grossly similar designs, although individual molecular components are specific to each one of the pathways. Ligands are secreted from restricted cellular sources and bind to receptor complexes on the receiving cells. Ligand–receptor binding activates a signaling cascade that ultimately leads to transcriptional regulation of target genes or, less often, to alternative non-transcriptional pathways when more immediate responses are needed. These signaling pathways are used over and over in development to regulate events as diverse as cell specification, proliferation, migration, and differentiation. It follows that their activity needs to be exquisitely controlled, ensuring that information among cells is activated where required and switched off at, or prolonged for, the appropriate time in order to obtain the required context-dependent output. There are different levels of regulation for these signaling molecules. Perhaps the most direct is the existence of classes of secreted proteins that interact with the ligand in the extracellular space, thereby preventing binding to their receptor. This occurs, for example, in the case of BMPs or Wnts, for both of which a large number of secreted antagonists exist ^1, 2^. Signaling enhancement also depends on secreted proteins that in some cases promote ligand diffusion as described for Wnt proteins ^3– 6^. In contrast, the currently known ligand-binding modulators of the Hh pathway are membrane-bound proteins, prompting the question of whether there is an advantage to such an organization.

Sonic hedgehog (Shh) is the most prominent member of the Hh family in vertebrates and one of the best examples of a classic morphogen ^7, 8^, as it induces the acquisition of specific identities in the receiving cells according to the levels and the duration of its signaling ^9^. Shh activates signaling with a mechanism that has been recently defined as “double-negative” ^10^. Indeed, in the absence of the ligand, its 12-pass transmembrane receptor Patched (Ptch) inhibits the seven-pass transmembrane GPCR (G-protein-coupled receptor)-like signal transducer Smoothened (Smo). In the absence of this inhibition, Smo would constitutively maintain the pathway active with the consequent transcription of Shh target genes, mediated by the family of Gli transcription factors. Shh binding to Ptch releases this inhibition and allows the expression of Gli-targeted genes. Gli targets include Ptch itself, thereby establishing a negative feedback loop, important also for limiting ligand dispersion ^9^. Thus, Ptch represses Shh pathway activation by controlling both ligand dispersion and the activity of the signal transducer. *In vitro* and *in silico* models have demonstrated that this organization confers robustness to the signaling gradient ^10^ and thus to Shh activity as a morphogen and likely to the additional functions that Shh exerts. So, in principle, there is an advantage to such an organization (see 11 for further discussion). However, activation of Shh signaling is modulated by other surface molecules that either contribute to Shh release from the producing cells, such as Disp (Dispatched) ^12^, or, on the receiving cells, interact with Ptch or Shh or both. The latter include Cdon (cell adhesion molecule-related, downregulated by oncogenes), Boc (Brother of Cdon), Gas1 (growth arrest protein 1) ^13, 14^, and Megalin/LRP2 (Megalin/low-density lipoprotein receptor-related protein 2) ^15^. The regulation of the membrane availability of Smo by the tetraspanin Atthog/Mosmo (modulator of Smo) is a recently described additional mechanism of Shh regulation ^16^. Is the presence of these membrane modulators also an advantage?

So far, no studies have formally addressed this question. Nevertheless, in this review, we will use the progressive formation of the vertebrate retina to discuss Shh functions in which some of these regulators have been implicated, pointing to potential advantages and unresolved or controversial issues.

## Cdon, Boc, Gas1, and LRP2 enhance Shh signaling during optic vesicles’ bilateralization

*Shh* is expressed along the entire axial mesoderm – anterior prechordal plate and posterior notochord – and the ventral midline of the vertebrate neural tube. This distribution prompted the use of the spinal cord as a primary model to understand the mechanism of Shh action ^17^. However, the progressive formation of the vertebrate retina offers an experimental paradigm with which to study how Shh is repurposed to shape multiple developmental aspects of the same structure, from early specification to connectivity.

The eyes are bilateral structures. Their neural component, the retina, originates from a group of cells, known as the retinal field, in the anterior neural plate. As the neural plate folds, cells of the retinal field become displaced laterally, forming two balloon-shaped optic vesicles at the side of the forming neural tube. *Shh* expression at the prechordal plate is critical for this initial morphogenesis: in the absence of Shh, optic vesicle bilateralism is lost and embryos form, in the most severe case, a single cyclopic eye or, in the milder cases, smaller eyes that are closer together. This phenotype, observed from humans to zebrafish ^18^, is part of a developmental anomaly known as holoprosencephaly (HPE), in which the ventral forebrain is not specified and the dorsal forebrain hemisphere tends to fuse together ^19, 20^. In amniotes, there are two concomitant events that contribute to optic vesicle lateralization. The first one is the Shh-dependent specification of the neural plate overlying the prechordal plate into the hypothalamic primordium, which therefore intervenes the two vesicles ^19^. The second is the patterning of the optic vesicles along their proximal–distal axis, which involves the Shh-mediated specification of the proximal/optic stalk domain (reviewed in 17). In teleost fishes, the Shh-mediated posterior-to-anterior migration of medial cells that intercalate into the retinal field is an additional factor ^21^. Genetic inactivation of basic components of the Shh pathway in mouse or zebrafish and mutational screening in patients with HPE confirmed the importance of Shh signaling in ventral central nervous system (CNS) patterning and thus in the proper positioning and growth of the optic vesicles ^18, 22^. Similar studies have also shown that *Cdon*, *Boc*, *Gas1*, and *LRP2* participate in these developmental events ^18, 23– 26^.

**Figure 1.**
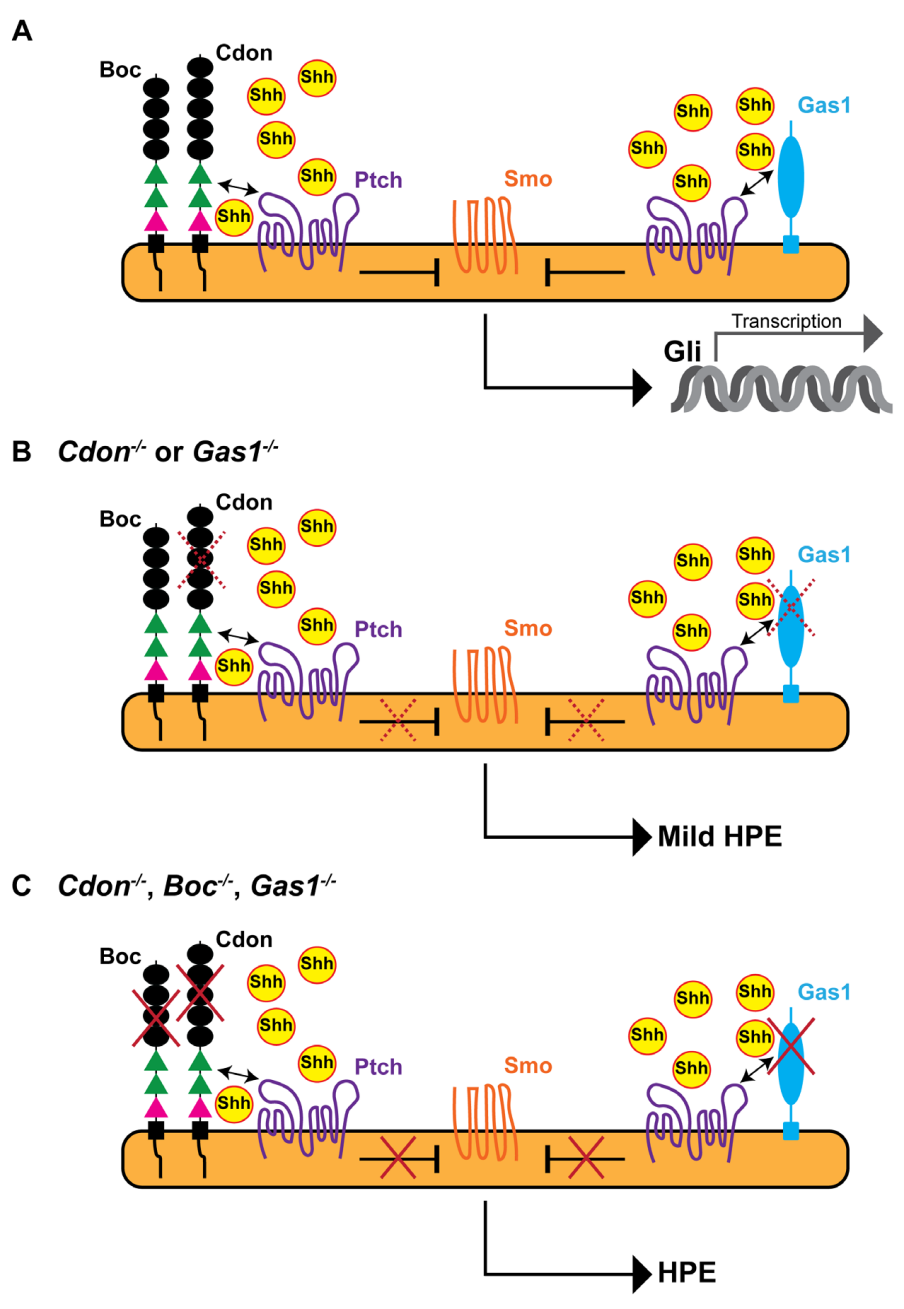
Cdon, Boc, and Gas1 act as positive regulators of Shh signaling during optic vesicle formation. The diagrams represent the interaction of Cdon, Boc, and Gas1 with Ptch and Shh during Shh-mediated patterning of the ventral neural tube in wild-type embryos ( **A**) or in embryos with genetic inactivation of either *Cdon* or *Gas1* function ( **B**) or lacking *Cdon*, *Boc*, and *Gas1* ( **C**). The three co-receptors interact with Ptch and the complex binds Shh with high affinity. In the presence of Shh, Smo is de-repressed (red crosses) and activates a signal transduction cascade that culminates with Gli-mediated transcription of Shh target genes. The Cdon/Ptch and Boc/Ptch interactions are mediated by the FnIIIa and FnIIIb domains (green) of Cdon and Boc, respectively. Binding of Shh to Cdon or Boc is mediated by the FnIIIc domain (pink). ( **B**) In the absence of either *Cdon* or *Gas1*, Shh is less activated (dotted red crosses), resulting in mild craniofacial defects. ( **C**) Loss of all three co-receptors prevents pathway activation, resulting in severe HPE, a phenotype that mimics *Shh* loss of function. Boc, Brother of Cdon; Cdon, cell adhesion molecule-related, downregulated by oncogenes; Gas1, growth arrest protein 1; HPE, holoprosencephaly; Ptch, patched; Shh, sonic hedgehog; Smo, smoothened.

Cdon and Boc are closely related cell adhesion molecules that can form homophilic and heterophilic complexes and interact with both Shh and Ptch (reviewed in 27). Cdon/Boc interaction with Ptch increases high-affinity ligand binding, indicating their function as Ptch co-receptors and thus as positive signaling regulators ^14, 23, 28– 30^. The two genes are expressed with largely overlapping patterns that include the entire dorsal neural tube and the developing eye and ear and the olfactory system ^31, 32^. This distribution often coincides with that of *Gas1* ^33^, encoding a GPI (glycosylphosphatidylinositol)-linked protein that also interacts with Shh and Ptch ^34, 35^ ( Figure 1A). Mouse embryos lacking *Cdon*, *Boc*, and *Gas1* show a phenotype that mimics *Shh* loss of function ^19^, which leads to the absence of the entire ventral neural tube resulting in severe HPE and early embryonic lethality ( Figure 1C). This indicates that the three co-receptors play positive and overlapping roles in regulating Shh pathway activation ^13, 14^. Furthermore, Shh signaling represses *Cdon*, *Boc*, and *Gas1* expression ^30, 36^. This suggests that these co-receptors may serve as buffers to prevent possible defects due to abnormally low Shh signaling because, if Shh activity decreases, their upregulation could boost signaling again. However, genetic inactivation of the individual co-receptor genes reveals non-equivalent roles. *Gas1* null mouse embryos present ventral neural tube defects, mild HPE, and mis-specification of the ventral retinal pigmented epithelium into a neural retina-like tissue ^33, 36^. *Cdon* null embryos display a similar mild HPE ( Figure 1B) with small eyes and coloboma (opened optic fissure) ^23, 37^. *Boc* null mice instead have none of these defects but, when crossed with either *Cdon* or *Gas1* mutants, enhance their respective HPE phenotype ^24, 25^. Whether Boc also modifies their respective specific eye phenotype remains to be studied. Somewhat in line with these differences, systematic genomic sequencing analysis of patients with HPE has identified causative mutations in the *CDON* gene ^23, 38^ but only sequence variations suggestive of a modifier role for *BOC* and perhaps for *GAS1* ^38^. Given that these co-receptors have all been shown to foster Shh signaling, it is not obvious why their loss of function causes these phenotypic differences, especially in the case of the closely related *Cdon* and *Boc*. One possibility is that Shh signaling exerts a differential negative regulation on their expression. Alternatively (or additionally), Cdon and Boc may employ distinct mechanisms to enhance Shh signaling, as recently suggested ^29^. For example, the ectodomain of Boc, but not that of Cdon, can be proteolyzed ^29^. If this proteolysis occurs *in vivo*, which is still a matter of speculation, Boc ectodomain could enhance Shh diffusion and at the same time terminate high-affinity binding of Shh to Ptch. The two effects may compensate one another, explaining the lack of HPE phenotype in *Boc* mutants. Variations in *Cdon*, *Boc*, and *Gas1* distribution may also underlie the observed differences in the mutants’ phenotype. This differential expression may also offer an alternative explanation for how Cdon and Boc influence Shh signaling. Indeed, whereas the ventral neural tube and optic vesicle expression of *Gas1* ^33^ makes it easy to understand its Ptch co-receptor function, the predominant dorsal expression of *Cdon* and *Boc* ^31, 32^ makes the same function less immediately understood. *Boc* and *Cdon* could be transiently expressed in the ventral neural tube right when needed for early patterning, as reported for the zebrafish *Boc* orthologue ^39^. However, the expression of *Cdon*, but not *Boc*, in the axial midline of both mouse and zebrafish ^30, 40^ suggests that Cdon could have the additional role of favoring Shh release from the producing cells. The *Drosophila* homologue of Cdon, interference Hh ( *ihog*), has been reported to have such an activity ^41^, although motif differences between Shh and its *Drosophila* homologue Hh call for caution in applying directly to vertebrates what has been learned in the fly ^42^. Nevertheless, the HPE phenotype of *Cdon* null embryos could easily be explained by an attenuated Shh release from the midline, a function in which *Boc* may not be implicated.

At the moment, this is only a hypothesis but it may be worth testing. It is equally unexplored whether LRP2 can functionally interact with Boc, Cdon, or Gas1 or with their possible different heterodimeric or trimeric complexes. LRP2 facilitates Shh/Ptch binding and promotes the internalization of the complex, which is required to relieve Smo inhibition. Thus, in the absence of LRP2, Shh signaling is impaired, leading to embryos with an HPE phenotype ^43^. It remains an open question whether LRP2 promotes Cdon, Boc, and Gas1 internalization when bound to Ptch or instead competes with them for Ptch and Shh binding.

## Cdon, Boc, and LRP2 can counteract Shh signaling during retinal development

**Figure 2.**
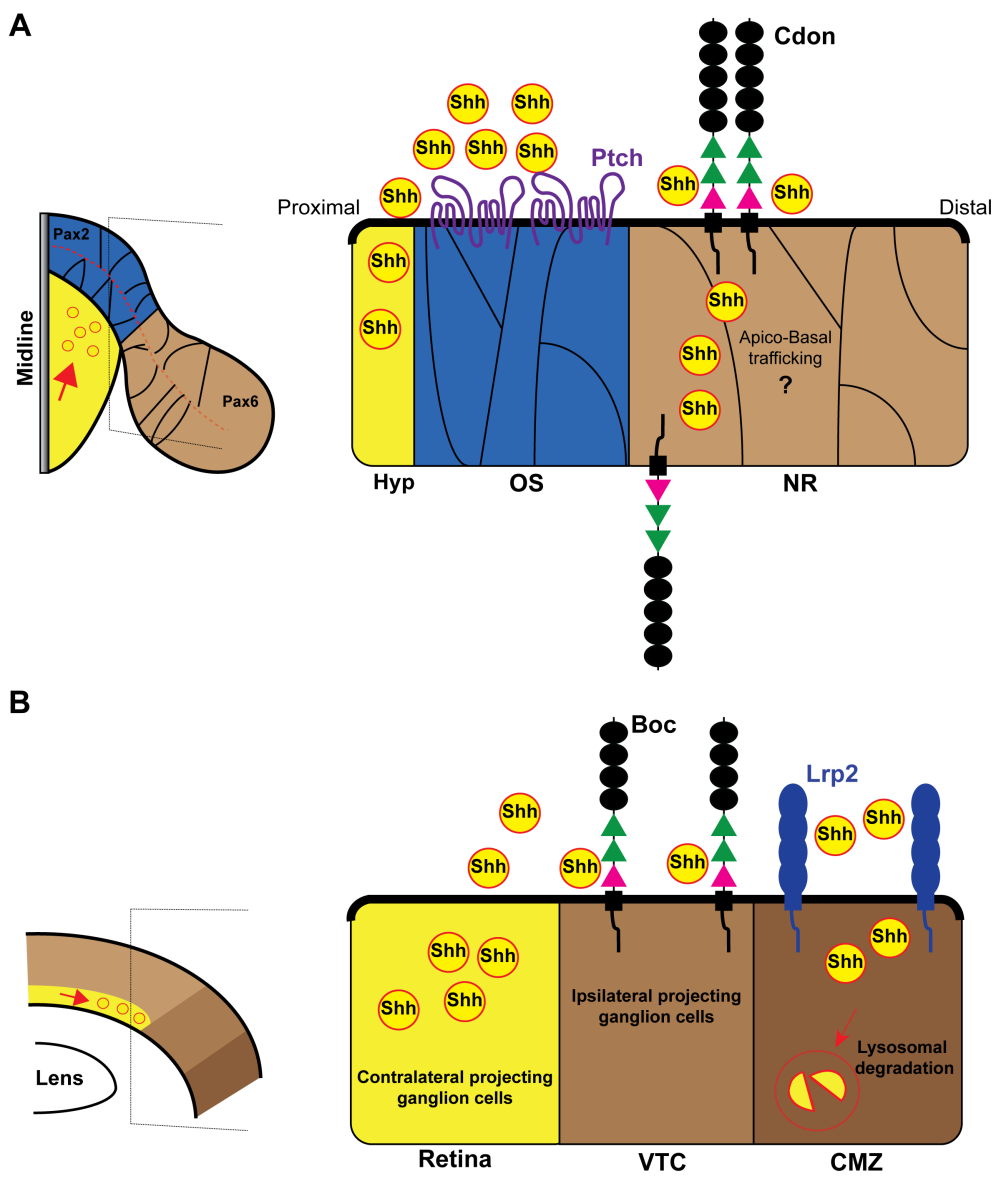
Cdon, Boc, and Lrp2 antagonize Shh activity during retina development. ( **A**) Schematic dorsal view of the optic vesicle (left) and enlarged view of the optic stalk/neural retina border (right). The expression domains of Pax2 (blue) and Pax6 (brown) are indicated in the scheme. At the border of these two domains, Cdon binds Shh, serving as a decoy receptor to protect the neural retina from midline-derived Shh activity. Note that the Ptch receptor localizes only in the Pax6-positive neural retina domain. ( **B**) Schematic frontal view of mature retina (left) and enlarged view of the retinal periphery (right). Contralateral RGCs produce and secrete Shh. Ipsilateral projecting RGCs express the co-receptor Boc that prevents Shh diffusion and thus signal activation. Low Shh signal allows for the specification of ipsilateral program specification in RGCs of the VTC. Lrp2/Megalin instead limits Shh proliferative activity by endocytic clearance of Shh at the CMZ. Boc, Brother of Cdon; Cdon, cell adhesion molecule-related, downregulated by oncogenes; CMZ, ciliary marginal zone; Hyp, hypothalamus; Lrp2, low-density lipoprotein receptor-related protein 2; NR, neural retina; OS, optic stalk; Pax2, paired box protein Pax-2; Pax6, paired box protein Pax-6; Ptch, patched; RGC, retinal ganglion cell; Shh, sonic hedgehog; VTC, ventrotemporal crescent.

The work we have discussed so far, independently of the still-puzzling aspects, supports a positive role of Cdon, Boc, Gas1, and LRP2 in Shh signaling and thus in the specification of the ventral CNS and eye separation. However, Cdon, Boc, and LRP2 have been shown to act as negative regulators of Shh signaling as retinal development progresses, although each one of them does so in different contexts ( Figure 2).

As mentioned before, the formation of two bilateral optic vesicles implies the compartmentalization of its neuroepithelium in different domains along the different axes. One of the first subdivisions occurs along the proximo–distal axis of the vesicle and originates the prospective optic stalk proximally and the prospective retina distally ( Figure 2A). The establishment of the optic stalk and retinal domains is defined by the specific and respective expression of two paired- and homeobox-containing transcription factors: paired box protein Pax-2 (Pax2) and Pax6 (reviewed in 44). The two factors cross-repress each other and thus define a sharp border between the two territories ^45^ ( Figure 2A). Shh signaling promotes *Pax2* expression, thereby imposing optic stalk identity. When Shh is reduced or absent, the optic stalk domain is smaller or absent and the two retinal domains tend to fuse together. Shh overexpression has the opposite effect with an excess of Pax2-positive optic stalk that overtakes the retinal domain by repressing *Pax6* ^17, 44^. This means that the right amount of Shh signaling is critical to form a precise boundary between the optic stalk and the retina. Recent studies have shown that, at least in zebrafish and chick embryos, Cdon participates in the establishment of this boundary ^40^. In both species, *ptch* is expressed in the *pax2*-positive optic stalk, whereas *cdon*, but not *boc*, is strongly expressed in the presumptive neural retina overlapping with *pax6* distribution ( Figure 2A) ^40^. The complementarity between *ptch* and *cdon* expression advocates against a synergistic role. Indeed, morpholino-mediated knockdown of *cdon* allows for the expansion of the optic stalk, decreases eye size, and prevents optic fissure closure, indicating that Cdon counteracts Shh effect ^40^. This phenotype depends on the ability of Cdon to bind Shh but not Ptch. Furthermore, it is a direct consequence of Cdon activity in the retina because targeted *cdon* overexpression in the zebrafish retina is sufficient to rescue the phenotype of *cdon* knockdown and spatiotemporal restricted interference with *Cdon* retinal expression in chick embryos mimics the zebrafish phenotype ^40^. The precise mechanism by which this happens is still unrefined; however, when mis-expressed close to the optic recess midline (a Shh source), Cdon binds Shh with great efficacy and serves as a sink to limit ligand availability to the nearby cells ^40^. This indicates that Cdon acts as a decoy receptor to protect the neural retina from Hh activity ( Figure 2A).

A similar function has been postulated for Boc during mouse retinogenesis ^46^. Retinal ganglion cells (RGCs) are the first neurons to be born in the retina of all vertebrates. Newly generated RGCs express Shh, and this expression promotes the propagation of RGC specification and differentiation, the proliferation of retinal precursors, and their differentiation toward other neuronal cell types (reviewed in 47, 48). In the mouse, a small proportion of RGCs located in the ventrotemporal crescent of the retina do not express Shh ^49^ ( Figure 2B). These neurons are special because, in contrast to all the Shh-positive RGCs, they project to the ipsilateral side of the brain, enabling the semi-binocular vision typical of rodents. These ipsilateral RGCs express Boc ^49, 50^. In these neurons, Boc is necessary to keep Shh signaling low, thereby enabling the expression of the transcription factor Zic2 ^46^, a determinant of the ipsilateral program ^51^. Thus, in *Boc* null mice, part of ipsilateral RGCs are mis-specified, acquiring a contralateral projecting phenotype with a consequent alteration of the retinal projections ^46^. In an additional and not necessarily contrasting view, Boc, present on the membrane of ipsilateral RGC growth cones, mediates guidance information provided by Shh at the optic chiasm midline, forcing the axons to enter the ipsilateral optic tract ^50^. Notably, Shh, transported along the axons of the contralaterally projecting RGCs ^49, 52^, seems to be released at high concentrations and with a still-unknown mechanism (see 53 for discussion), right at the chiasm providing Boc-mediated repulsive information to ipsilateral axons ^52^. Thus, in this case, Boc would act as a positive mediator of Shh. Whether the same molecule can have a double function in the same cell remains to be established, but, in a speculative view, Boc interactions at the perikaryon could be different from those existing at the growth cone.

Independently of this still-unanswered question, both Cdon and Boc can function as negative regulators of Shh activity, limiting ligand dispersion, a function that has been observed in *Drosophila* wing disc and ovary development ^41, 54, 55^. Incidentally, both Cdon and Boc, when ectopically expressed close to a Shh source, localize predominantly at the basal side of the neuroepithelial cells, where they accumulate most of the bound Shh protein ^40^. A recent study revisiting the function of Hhip (Hh-interacting protein)—initially defined as a membrane-bound negative regulator of HH signaling ^56^—showed that Hhip is secreted and localizes to the neuroepithelial basal membrane ^57^. The basal localization of both Cdon and Hhip is interesting because it may serve to clear the ligand from the apical surface of the neuroepithelial cells, where the primary cilium localizes. This organelle is fundamental for Shh signal transduction, as it hosts the main components of the transduction machinery of this pathway ^58^.

A negative regulation of Shh signaling, based on a different mechanism of ligand clearance, has also been proposed for LRP2. Though initially expressed in the whole optic cup, LRP2 expression becomes restricted to the peripheral margin as retina differentiation proceeds. This region, called the ciliary marginal zone (CMZ), is a source of progenitor cells in fish and amphibians ^59^ and likely also in the mammalian embryonic retina ^60, 61^. The CMZ is normally devoid of Shh activity. Deficiency of *Lrp2* in mice or zebrafish causes enlarged and exophthalmic eyes ^62– 65^, a pathological condition known as buphthalmos and observed in patients carrying *LRP2* mutations ^66^. Searching for an explanation for this phenotype, Christ *et al*. ^65^ found elevated transcript levels for GLI family zinc finger 1 ( *Gli1*) and *Ptch1* genes, two Shh targets, suggesting that LRP2 protects the CMZ from the influence of RGC-derived Shh. In the absence of LRP2, Shh induces CMZ progenitor hyperproliferation, expanding the overall eye size. Mechanistically, LRP2 mediates lysosomal clearance of Shh alone, thereby maintaining the CMZ quiescent ^65^ ( Figure 2B).

Although their local function has not been explored in detail, *Cdon*, *Boc*, and *Gas1* are also strongly expressed in the CMZ ^36, 40, 49^, making this structure an attractive model to study possible interaction among all these Shh-binding proteins. In a speculative view, they could all concur to make the CMZ a Shh-free zone given that Gas1 has also been initially proposed to work as a Shh sink ^34^.

In conclusion, going back to the original discussion point, “economy” could be the main advantage of controlling potent signaling molecules with membrane-bound proteins. Cdon, Boc, and Lrp2—in the specific case of Shh—seem to act as both positive and negative regulators of the signaling pathway depending on their additional interaction with other membrane-bound proteins (Ptch). In a speculative view, an economical way of changing the role for these regulators from a positive to a negative one might be their shuttling from the apical to the basal membrane. The destiny of Shh bound to Boc or Cdon when these proteins act as negative regulators is a matter of speculation. However, in the same economical view, unwanted Shh could be recycled back to the tissue where it is needed, such as from the neural retina to the optic stalk. The observed redundancy of regulatory molecules may not fall into the view of economy, although redundancy might be the best way of ensuring the needed levels of Shh during development and homeostasis.

## Abbreviations

Bmp, bone morphogenetic protein; Boc, Brother of Cdon; Cdon, cell adhesion molecule-related, downregulated by oncogenes; CMZ, ciliary marginal zone; CNS, central nervous system; Gas1, growth arrest protein 1; Hh, hedgehog; Hhip, hedgehog-interacting protein; HPE, holoprosencephaly; LRP2, low-density lipoprotein receptor-related protein 2; Pax2, paired box protein Pax-2; Pax6, paired box protein Pax-6; Ptch, patched; RGC, retinal ganglion cell; Shh, sonic hedgehog; Smo, smoothened
